# Changing patterns of cardiovascular diseases and cancer mortality in Portugal, 1980–2010

**DOI:** 10.1186/1471-2458-12-1126

**Published:** 2012-12-29

**Authors:** Marta Pereira, Bárbara Peleteiro, Simon Capewell, Kathleen Bennett, Ana Azevedo, Nuno Lunet

**Affiliations:** 1 Department of Clinical Epidemiology, Predictive Medicine and Public Health, University of Porto Medical School and Institute of Public Health of the University of Porto (ISPUP), Al. Prof. Hernâni Monteiro, Porto, 4200-319, Portugal; 2 Department of Public Health and Policy, University of Liverpool, Liverpool, UK; 3 Department of Pharmacology and Therapeutics, Trinity Centre for Health Sciences, St James’s Hospital, Dublin, Ireland

**Keywords:** Cancer, Cardiovascular diseases, Mortality, Trends, Portugal

## Abstract

**Background:**

Cardiovascular diseases and cancer are jointly responsible for more than half all deaths in Portugal. They also share some important risk factors and act as mutual competing risks. We aimed firstly to describe time trends in death rates and years of life lost due to cardiovascular diseases and cancer in the Portuguese population from 1980 to 2010; and secondly to quantify the contribution of the variation in population and age structure, and age-independent “risk” by cardiovascular or oncological causes to the change in the corresponding number of deaths.

**Methods:**

We estimated the annual percent change in age-standardized mortality rates from cardiovascular diseases and cancer, in each sex. The specific contribution of demographic changes (due to changes in population size and in population age structure) and the variation in the age-independent “risk” of dying from the disease to the observed trends in the number of deaths was quantified using the tool RiskDiff. Years of life lost were computed using the Global Burden of Disease method.

**Results:**

Among men, the mortality rate from all cardiovascular diseases was more than two-fold higher than cancer mortality in 1980. However, three decades later mortality from cancer surpassed cardiovascular diseases. After 2005, the years of life lost from cancer were also higher than from cardiovascular diseases. Among women, despite the decrease in death rates, cardiovascular diseases remained the leading cause of death in 2010 and their absolute burden was higher than that of cancers across the whole period, mainly due to more events in older women.

**Conclusions:**

In Portugal, the 20th century witnessed a dramatic decrease in the cardiovascular disease mortality and YLL, and the transition towards cancer. In more recent years, the highest burdens of disease came from cancers in men and from cardiovascular diseases in women.

## Background

In 2004, cardiovascular diseases (CVD) were the leading cause of death in the world, responsible for approximately 32% of all deaths in women and 27% in men. After infectious and parasitic diseases, cancer ranked third, causing some 12% of all deaths among women and 13% among men [1]. Taking the age at death into account, however, CVD were responsible for only 14% and cancer for 8% of the total years of life lost (YLL) in 2004, surpassed by infectious and parasitic diseases, perinatal conditions and unintentional injuries.

Age-standardized mortality rates from CVD have been declining for several decades in Europe [2,3], while the downward trend in mortality from some common cancers is more recent, and varies with the type of cancer. The overall cancer mortality has been decreasing less steeply, and in some Eastern European countries is still increasing [4]. While the proportion of all deaths from CVD was almost three-fold higher than from cancer in Europe, the YLL were less than two-fold higher for CVD than cancer, in 2004 [1,5].

In Portugal, CVD were the leading cause of death in 2006, followed by cancer, and these two groups of diseases were responsible for more than half of all deaths [6]. Portugal is characterized by a very higher contribution of cerebrovascular disease to mortality relative to coronary heart disease [1] and long-standing higher systolic blood pressure, as compared to most other European countries [7]. The types of cancers that cause more deaths in Portugal are lung and stomach among men and breast among women [4]. The relatively early stage of Portugal in the smoking epidemic results in an expected increase in the smoking-related burden of disease and deaths, particularly among women [8,9].

We aimed to describe time trends in the absolute number of deaths, death rates and YLL from CVD and cancer in the Portuguese population, during the period 1980–2010, and to quantify the contribution of the variation in the population’s size and age structure, and age-independent “risk” of death by cardiovascular or oncological causes to the change in the corresponding number of deaths.

## Methods

### Sources of data

The number of deaths from all CVD [International Classification of Diseases 10th revision (ICD 10): I00-I99; 9th revision (ICD 9): 390–459] and all malignant neoplasms**,** hereafter just referred as cancer (ICD 10: C00-C99; ICD 9: 140–239), as well as the estimates of the population at risk in each year, were obtained from official statistics [10,11].

All data were obtained from 1980 to 2010 for each sex in age groups (<1, 1–4, 5-year age groups up to 80–84 and ≥85 years).

### Trends in mortality rates and years of life lost

Standardized mortality rates were computed by the direct method using the European standard population as reference [12]. We performed a joinpoint regression analysis, using Joinpoint® version 3.4 from the Surveillance Research Program of the US National Cancer Institute [13], to calculate the annual variation in mortality and to identify points of significant change in the log-linear slope of the trend (joinpoints) [14]. The analysis starts with the minimum number of joinpoints (no joinpoints corresponds to a straight line), and tests whether one or more joinpoints significantly improve model fit. The minimum number of observations from a joinpoint to the earliest or the latest years and between two joinpoints was set to 5. We present the results of best fitting models for the trends in men and women. The estimated annual percent change (APC) in mortality for each period was calculated assuming a Poisson distribution and taking the calendar year as the independent variable.

The analyses of the trends in the mortality rates and numbers of deaths were performed for all ages and by age groups (0–14, 15–34, 35–54, 55–74 and ≥75 years).

The YLL due to premature mortality for each cause (CVD and cancer), gender and age group were computed using the Global Burden of Disease method [15] by multiplying the number of deaths at each age by the life expectancy at the age at which death occurs. We considered the recommended standard life expectancy at birth of 80 years for men and 82.5 for women. The average age at death was set to the mid-point of each five-year age group, apart from the infant deaths (where it is assumed to be 0.1 years in low mortality countries), the 1–4 year age group (assumed to be 2.6 years) and the oldest group (assumed to be 87.5 years) [15]. We applied a 3% time discount rate to assign less weight to the YLL corresponding to the periods more distant to the moment of death than to those referring to the first years after death, an age-weighting parameter to weigh YLL in very young and old ages less than other ages (Global Burden of Disease standard value is 0.04) and an age-weighting correction constant so that the introduction of age-weights did not alter the total number of YLL (Global Burden of Disease standard value is 0.1658) [15]. The total YLL for each cause and gender was obtained by adding the YLL of all age groups.

### Contribution of changes in demographics and age-independent “risk”

We used the tool RiskDiff, a web-based application from the Catalan Institute of Oncology to assess the specific contribution of demographic changes (due to changes in population size and in population age structure) and the variation in the age-independent “risk” of dying from the disease to the observed trends in the number of deaths [16]. This analysis was performed for the periods with constant log-linear trend identified in the joinpoint analysis. RiskDiff outputs the results over an entire time period. To allow comparisons among intervals of different length, we estimated annual effects assuming geometric change over time [17].

## Results

### Trends in mortality rates and years of life lost

**Figure 1:**
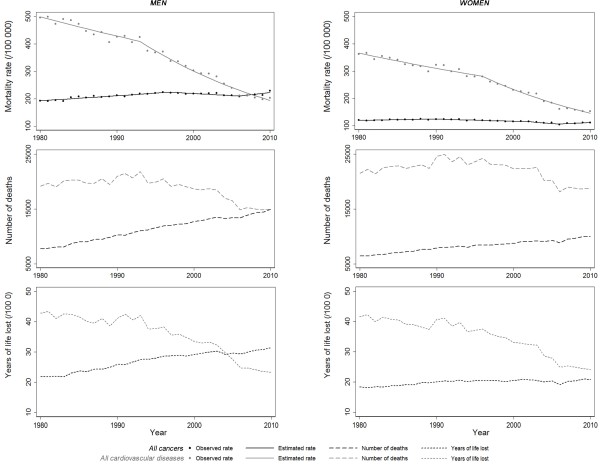
Time trends in cardiovascular diseases and cancer 1980–2010, by sex: age-standardized (European population) mortality rates, number of deaths and years of life lost.

Among men, the age-adjusted mortality from CVD decreased between 1980 and 2010 (Figure 1 and Table 1). The decrease was more pronounced after 1993, with an APC of −4% per year *versus −*1.5% per year between 1980 and 1993. The age-adjusted mortality rate from cancers increased 0.9% per year between 1980 and 1997, then declined slightly, 0.7% per year, until 2006 and increased 1.5% per year thereafter, in men. Among women, the pattern of the age-adjusted mortality from CVD was similar to that of men, decreasing between 1980 and 2010, more pronouncedly after 1996, reaching an APC of −4.6% per year. The mortality rate from cancer increased from 1980 to 1990, then decreased slightly until 2006 (more steeply between 2002 and 2006, by 2% per year), and then increased 1.3% per year until 2010.

**Table 1:** Annual change and contribution of demographic changes and age-independent “risk” to cardiovascular diseases and cancer mortality 1980–2010, by sex

	**Disease**	**Annual change in**		**Annual variation in number of deaths due to variation in**		
	Period *	**Age-standardized mortality rate % (95% CI)**	**Number of deaths%**	**Population size%**	**Population age structure%**	**Age-independent “risk”%**
**Men**	**All cardiovascular diseases**					
	1980-1993	−1.5 (−2.0 to −1.0)	1.00	0.15	2.08	−1.59
	1993-2010	−4.3 (−4.7 to −3.9)	−2.20	0.18	1.35	−5.26
	**All cancers**					
	1980-1997	0.9 (0.7 to 1.1)	2.76	0.28	1.65	1.16
	1997-2006	−0.7 (−1.2 to −0.2)	1.18	0.60	1.32	−0.78
	2006-2010	1.5 (0.2 to 2.9)	2.85	−0.26	0.66	2.47
**Women**	**All cardiovascular diseases**					
	1980-1996	−2.0 (−2.3 to −1.6)	0.75	0.17	1.98	−1.94
	1996-2010	−4.6 (−5.1 to −4.2)	−1.79	0.23	2.00	−5.92
	**All cancers**					
	1980-1990	0.4 (0.2 to 0.7)	2.09	0.18	1.36	0.65
	1990-2002	−0.6 (−0.9 to −0.4)	1.19	0.37	1.32	−0.61
	2002-2006	−1.9 (−3.4 to −0.5)	−0.62	0.41	1.26	−2.39
	2006-2010	1.3 (0.4 to 2.3)	3.17	−0.10	1.08	2.24

* We considered the periods with constant log-linear trend identified in the joinpoint analysis.

Among men, in 1980 the mortality rate from CVD was more than two-fold higher than that from cancer, but in 2008 the mortality rate from cancer surpassed the mortality rate from CVD, due to opposite trends between the two groups of diseases since the mid-2000s (Figure 1 and Table 1); the total number of deaths from CVD was higher than from cancer between 1980 and 2010, but converged to around 15000 deaths each in 2010. Among women, despite the convergence in the mortality rates from both diseases, CVD remained the leading cause of death in 2010. Between 1980 and 2010, the burden of CVD was much higher compared to cancer and, in 2010, CVD were responsible for 10,000 deaths more than cancers.

Among men, the YLL from cancer increased between 1980 and 2010, while the YLL from CVD decreased, mainly after the mid-1990s (Figure 1); in 2005, the YLL from cancer surpassed those from CVD and were clearly higher thereafter. Among women, the trends in YLL from CVD and cancer were very similar to those observed for mortality rates.

**Figure 2:**
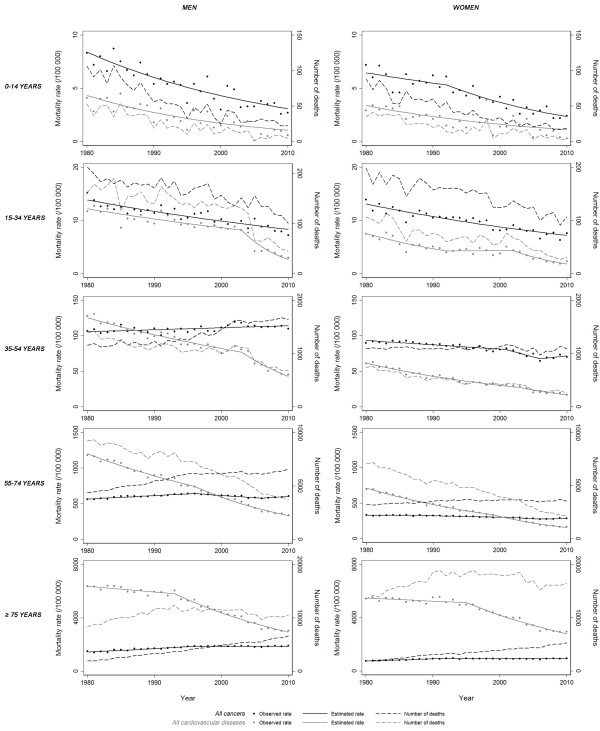
Time trends in cardiovascular diseases and cancer 1980–2010, by sex: mortality rates and numbers of deaths, by age groups.

When analyzing trends by age groups, the mortality rate from CVD decreased between 1980 and 2010, for all age groups among men, while a decrease in the mortality rates from cancer was only observed for the younger age groups (up to 35 years) (Figure 2). Cancer was a cause of death more frequently than CVD among those under 35 years of age between 1980 and 2010, in the last two decades for those aged 35 to 54 years and in the last decade for those aged 55 to 74 years. Cardiovascular diseases were the main of these two causes of death from 1980 to 2010 among men aged over 75 years. Among women, the mortality rate from CVD decreased between 1980 and 2010 in all age groups, while mortality rates decreased in younger women, with a recent attenuation for ages 35–54 years, and remained approximately constant in the two older age groups (Figure 2). Cancer was the main cause of death until 55 years between 1980 and 2010 and in the last decade in the age group 55–74 years, while CVD were always the main cause of death in women over 75 years.

### Contribution of changes in demographics and age-independent “risk”

Between 1980 and 2010, the increase in population size and changes in the age structure towards a higher proportion of older subjects contributed to an increase in the number of deaths by CVD (around 0.2% per year and 2% per year, respectively) (Table 1). Similarly, between 1980 and 2006, the increase of the population size contributed to an increase in the number of cancer deaths [between 0.2% per year in women (1980–1990) and 0.6% per year in men (1997–2006)], as did the ageing of the Portuguese population (1.6% per year in men from 1980 to 1997 and 1.3% per year in men from 1997 to 2006 and in women overall). In the period 2006–2010, the Portuguese population decreased in number, thereby contributing to a decrease in number of deaths by cancer (less than 0.3% per year), while the population ageing contributed to the change in the cancer mortality rates by an annual variation of 0.7% in men and 1.1% in women (Table 1).

The age-independent “risk” of death from CVD decreased between 1980 and 2010, more significantly since the mid-1990s, after which point it translated into a decrease in the total number of deaths of around 2% per year. After declining for several years, the risk of death from cancers increased by more than 2% per year from 2006 to 2010. Overall, the number of cancer deaths increased in both sexes between 1980 and 2010, except for a short period between 2002 and 2006, among women (Table 1).

## Discussion

We observed a dramatic decrease in Portuguese CVD mortality rates from 1980 to 2010 in both sexes. However, this decrease was reflected in a fall in the total number of deaths only since the mid-1990s, due to the increases and ageing of the population. For cancer, we observed a much smaller decrease in the mortality rates from the 1990s until 2006 in both sexes, and an increase thereafter. These trends, together with increased premature mortality from cancer, meant that in men, YLL from cancer surpassed those from CVD after 2005.

In recent years, cancer has become the leading cause of death among men, in many other European countries, such as France, Spain, Netherlands and Italy [18,19]. Cardiovascular diseases and cancer interact as mutually competing risks. The observed decreasing trends in CVD mortality may have contributed to the recent increase in mortality rates from cancer, with more individuals surviving at risk of cancer. We also observed a decrease in the number of deaths caused by gastro-intestinal diseases, injuries and poisoning, and infectious and parasitic diseases in the last three decades, in Portugal [10]. Since some of these causes of death are more common in the young and middle-aged population, these trends leave more at risk for the diseases associated with older age, such as diabetes, CVD or cancer [20].

The mortality trends from CVD in Portugal paralleled steady mortality declines in Western Europe, North America, Japan and other developed areas of the world [2,3]. This reflects decreases in both cerebrovascular and ischaemic heart diseases, the two major contributors to this group and more frequent in the older population [3,21]. In Portugal, cerebrovascular disease has been the main cause of death during the last decades [6,22]. In developed countries, the mortality rates from stroke have been decreasing more steeply in countries with higher departure rates, which is the case of Portugal [23]. This could reflect general improvements in living conditions, lower salt consumption thanks to improved methods for food preservation and recently, perhaps, lower case-fatality due to stroke units and better emergency care [24]. Regarding ischaemic heart disease, modeling studies in many developed countries consistently suggest that 45% to 75% of the mortality declines can be attributed to decreases in major risk factors, with the remaining attributable to medical and surgical treatments [25-28]. In Portugal, we likewise observed notable decreases in the mean systolic blood pressure and blood cholesterol, smoking consumption among men, and some dietary modifications, accompanied by the increased use of therapies for acute coronary syndromes [7,9,29,30]. Deaths from CVD in younger age groups probably reflected improved diagnostic capacities and successful early surgical interventions for congenital heart disease, plus better medical care for hypertension, cardiac arrhythmias and endocarditis [31]. The pattern in the trends of YLL suggests that death by CVD is being increasingly delayed, clearly affecting mostly the elderly population.

Interpreting trends in cancer mortality rates can be challenging, because they reflect trends in several common cancers. In Portugal, the specific cancers that could particularly contribute to the slight decrease in overall cancer mortality rate between the 1990s and 2006 among adults include stomach cancer (in both sexes), prostate and lung among men and breast and uterus among women [4]. Despite the trends in mortality rates, the total number of deaths and YLL have increased steadily. Among the more frequent cancers stomach, lung and breast cancer are associated with death at relatively younger ages [20], contributing more to the increase in YLL due to cancer. On the other hand, we expected a lower contribution to YLL from prostate cancer, since this is a disease mainly of the elderly population [20]. In younger ages, the decrease in leukaemia may also contribute [4]. The few adult cancers increasing in more recent years differ between women and men. In women, the very recent increase in breast cancer mortality plus the long-term increase in the lung cancer mortality could help explain the turning point observed in the cancer mortality rate and YLL in 2006 [32]. In men, colorectal cancer is the only common cancer which is increasing [10]. However, further close analyses during the forthcoming years are clearly necessary to confirm that the trends observed in the 4 years since 2006 persist longer term. The steady decline in cancer mortality is likely to reflect improvements in risk factors such as male smoking and diet, compounded by better access to earlier and specialized diagnosis, staging and treatment [33]. Worryingly, the mortality for some cancers has increased since 2006, which may reflect an increase exposure to other risk factors, such as obesity [34]. The transition to a more Westernized diet has occurred faster in Portugal than in some other Mediterranean countries [35]. The continued increases in smoking among women across the Eastern, Western and Southern parts of Europe [36] are very concerning. Unless halted, these will inevitably increase cancer mortality among women in Portugal. Furthermore, the late implementation and the low participation rate in organised cancer screening programmes [37] may delay the potential benefits. In 2008, the breast and cervical cancer screening did not cover the entire country and the screening program for colorectal cancer was still in its infancy [37]. Apart from the variation in the population structure, we identify an increase in the risk of dying from cancer after 2006, which inverts the previous decrease observed in the 1990s.

The interpretation of the reported trends depends on the quality of the source data. Misattribution or miscoding can occur mainly because of incorrect diagnoses, incorrect or incomplete death certificates, misinterpretation of ICD rules for coding underlying causes, and variations in the use of categories for unknown and ill-defined causes [38]. Like most developed countries Portuguese mortality data have high coverage, but have been considered to have low quality regarding the high proportion (21%) of deaths coded as ill-defined causes [38]. Additionally, and more specifically, there is evidence of inaccuracies in attribution of frequent causes of death. For instance, in a validation study in the mid-2000s, stroke was not confirmed in a large proportion of death certificates indicating it as the underlying cause of death, and 5% of patients were identified only after the review of death certificates [39]. The accuracy in attribution to causes of death varies over time due to quality of diagnoses and also due to changes in the coding rules. In Portugal, two revisions of the ICD were used in the calendar period considered in this study, with ICD-10 replacing ICD-9 in 2002. However, since the ICD-10 is more detailed than ICD-9 and we used two large groups of diseases, without focusing on very specific codes, errors might have been minimised.

Although we do not have information on CVD incidence and case-fatality in this population, we predict that death from CVD will be mainly associated with old age in Portugal, as in highly developed Western European countries. However, the prevalence of CVD is likely to increase and so is the related demand for healthcare. The observed trends in cancer mortality highlight the importance of primary and secondary prevention, demanding better planning in health services, in order to obtain improvements in earlier detection and/or increasing proportions of patients receiving new or more aggressive treatment.

## Conclusions

The 20th century witnessed a dramatic decrease in cardiovascular disease mortality and YLL in Portugal and a recent transition towards a higher burden of cancer. Furthermore, the sustainability of gains against CVD is threatened by recent trends in obesity among men and women and increasing smoking among women. These same risk factors play an important causal role in several cancers, highlighting the importance of future prevention strategies. This analysis of the mortality data taking into account demographic effects, produced results that are easily usable for policy makers. The data on the absolute number of cases and demographic determinants is potentially highly relevant for planning purposes and also for predicting future needs.

## Abbreviations

CVD: Cardiovascular diseases; YLL: Years of life lost; ICD: International Classification of Diseases; APC: Annual percent change.

## Competing interests

The authors declare that they have no competing interests.

## Authors’ contributions

MP, collaborated in the acquisition, analysis and interpretation of the data, and wrote the first draft of the article. BP, collaborated in the in the acquisition, analysis and interpretation of the data, and in the revision of the article. SC, collaborated in the interpretation of the data and reviewed the article critically for important intellectual content. KB, collaborated in the interpretation of the data and reviewed the article critically for important intellectual content. AA, designed the study, analysed and interpreted the data, and reviewed the article critically for important intellectual content. NL, designed the study, analysed and interpreted the data, and reviewed the article critically for important intellectual content. All authors read and approved the final manuscript.

## Pre-publication history

The pre-publication history for this paper can be accessed here:

http://www.biomedcentral.com/1471-2458/12/1126/prepub
